# Supplementary material for: Structural basis of SALM5-induced PTPδ dimerization for synaptic differentiation
Source: Nat Commun. 2018 Jan 18;9:268. doi: 10.1038/s41467-017-02414-2 (PMC5773555; doi:10.1038/s41467-017-02414-2)
Supplement: Supplementary file 1 — Supplementary Information [file 41467_2017_2414_MOESM1_ESM.pdf]

Supplementary Information

Supplementary figures

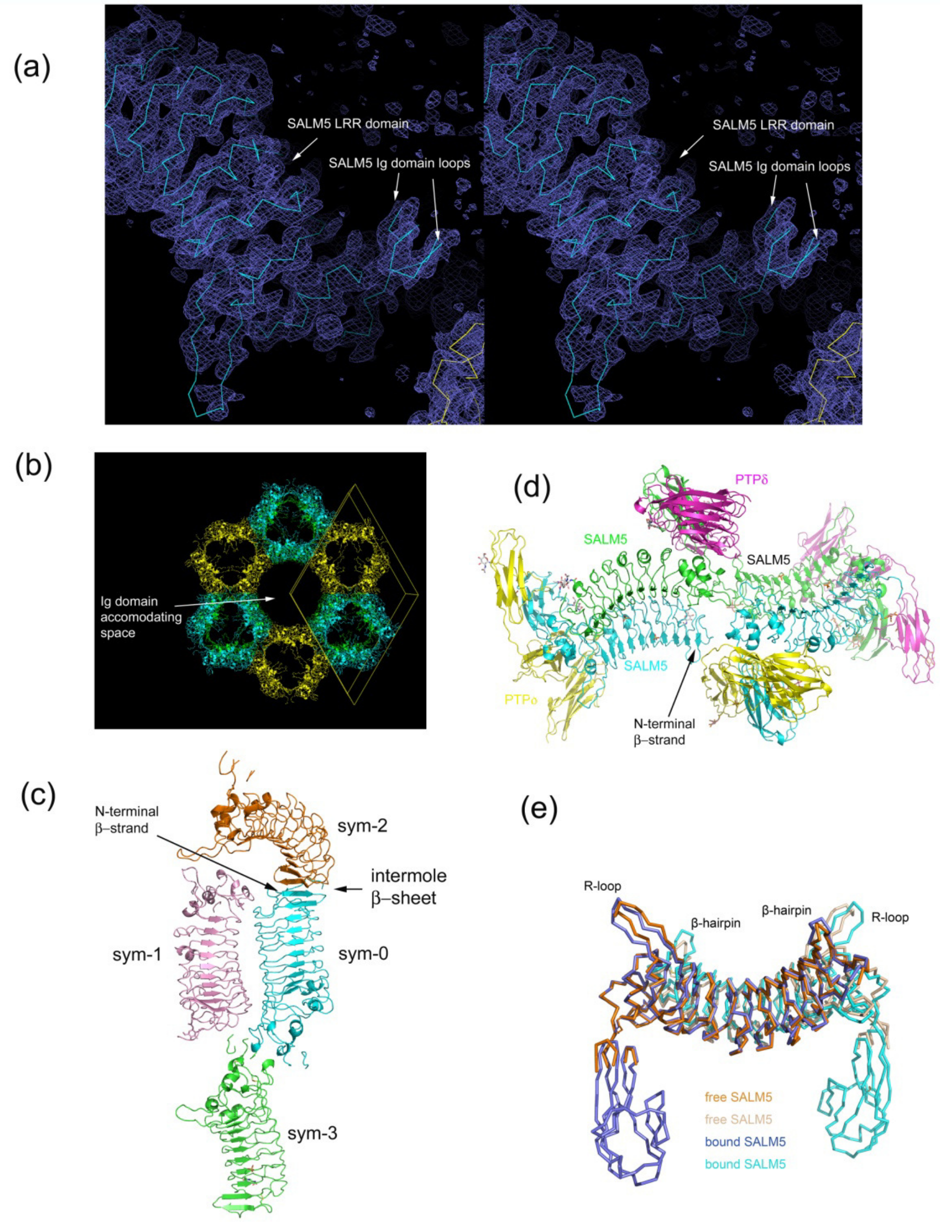

Supplementary Figure 1: Crystallographic analysis of human SALM5 LRR-Ig (labeled as SALM5 for short in this figure) alone and in complex with human PTPδ Ig 1-3 (MeA^{-}) (labeled as PTPδ in this figure). (a) Cross-eye stereo view of sigma-A

weighted $2F_o$-F_c density map (contoured at 1.0 σ) for one SALM5 molecule (in cyan) and its symmetry mate (in yellow). Only two loops in SALM5 Ig domain could be discerned in the density. (b) Crystal packing of free SALM5 viewed along a crystallographic 6_3 axis. The "missing" majority of SALM5 Ig domain should be accommodated in the sphere-like space. (c) In the crystal, a SALM5 molecule, labeled as sym-0 (represented in cyan ribbon), packed with three symmetry mates sym-1 (pink), sym-2 (orange) and sym-3 (green). Sym-0 and sym-1 formed an anti-parallel side-by-side dimer. Noteworthy, an intermolecular β-sheet formed between sym-0 and sym-2 through their N-terminal β-strands. (d) Crystal packing analysis of the SALM5/PTPδ complex in ribbon representation. SALM5: green or cyan; PTPδ, yellow or pink. The side-by-side dimerization mode of SALM5 is still observed. However, the N-terminal β-strand of SALM5 didn't form an intermolecular β-sheet. (e) Superimposition of a non-crystallographic 2-fold axis related SALM5 dimer (bound with PTPδ, in cyan or slate) with a crystallographic 2-fold axis related SALM5 dimer (alone, in orange or salmon). The structures of the two dimers can be aligned well with an RMSD of 0.69 Å for 266 paired C_α atoms.

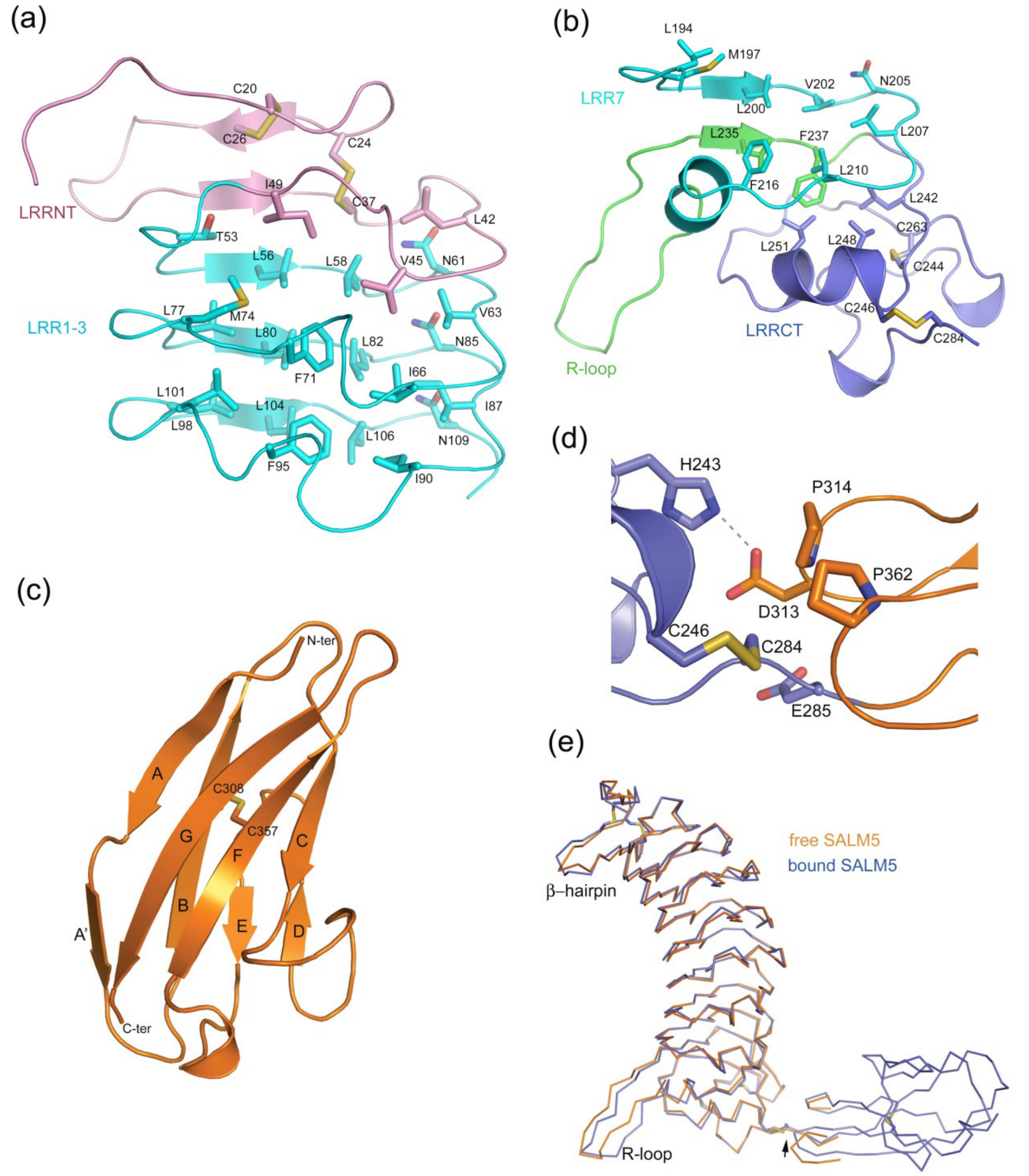

Supplementary Figure 2: Structural analysis of the SALM5 monomer. (a) Top view of the N-terminal half of the SALM5 LRR (in ribbron) including LRRNT subdomain (pink) and LRR 1-3 (cyan). The characteristic residues that determine the topology of a leucine-rich repeat are shown in sticks. (b) Ribbon representation of LRR7 (cyan), R-loop (green) and LRRCT (slate). Key residues are shown in sticks. (c) Ribbon representation of Ig domain with a 4-on-4 topology (sheet ABED versus A′GFC). Disulfide bonds, cross-linking the two sheets, are indicated as sticks. (d) Close-up

view of residues on the interface between LRR (slate) and Ig (orange) domains. A hydrogen bond formed between H243 and D313 is indicated as a dot line. (e) Superimposition of free SALM5 with PTPδ-bound SALM5. Domain rotation (as indicated in an arrow) could occur around the linker between LRR and Ig domain. Different from the LRRs 2-6 that belong to a typical 24-residue LRR repeat, the LRR7 is composed of 25 residues and folds into a short helix at its C-terminal fragment, as seen in panel (b). Hydrophobic residues L210 and F216 of LRR7, L235 and F237 of the R-loop motif, and L242, L248 and L251 of the LRRCT subdomain cluster together, stabilizing the configuration of the C-terminal portion of the solenoid. As shown in panel (d), the interaction between LRR and Ig domains is mainly governed by a few van der Waals contacts engaged by H243, C246 and C284 in the LRRCT subdomain and D313, P314 and P362 on the N-terminal end of the Ig domain, as well as a hydrogen bond formed between H243 and D313. Such a weak interdomain interaction suggests that the SALM5 Ig domain would have a variable orientation relative to the LRR domain, therefore explaining the lack of density for the Ig domain in the crystal structure of free SALM5. Superimposition of the C_{α} atoms of free and PTPδ-bound SALM5, as displayed in panel (e), highlights a potential dynamic movement of the Ig domain around the linker, which is confirmed by the absence of the majority of the Ig domain in free SALM5 structure.

(a)

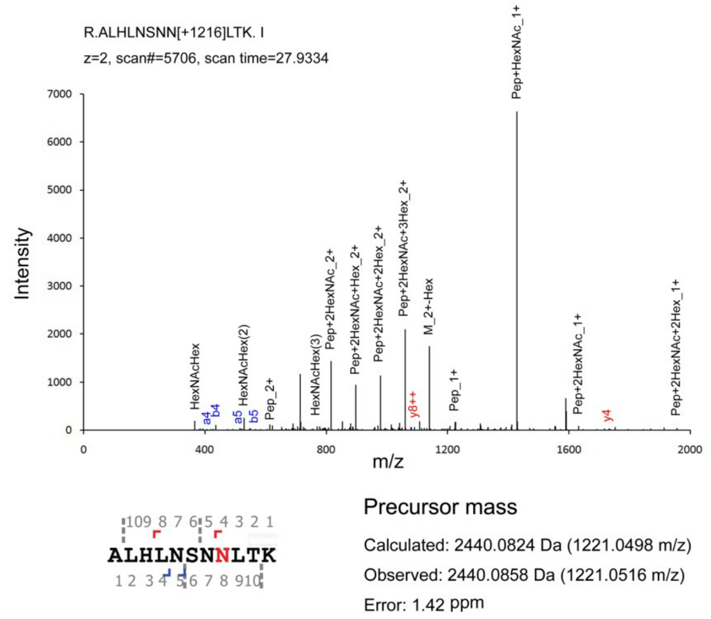
R.ALHLNSNN[+1216]LTK. I
z=2, scan#=5706, scan time=27.9334
Intensity
m/z
HexNAcHex
HexNAcHex(2)
HexNAcHex(3)
Pep_2+
Pep+2HexNAc_2+
Pep+2HexNAc+Hex_2+
Pep+2HexNAc+2Hex_2+
Pep+2HexNAc+3Hex_2+
M_2+-Hex
Pep_1+
Pep+HexNAc_1+
Pep+2HexNAc_1+
Pep+2HexNAc+2Hex_1+
a4
b4
a5
b5
y8++
y4
ALHLNSNNLTK
Precursor mass
Calculated: 2440.0824 Da (1221.0498 m/z)
Observed: 2440.0858 Da (1221.0516 m/z)
Error: 1.42 ppm

(b)

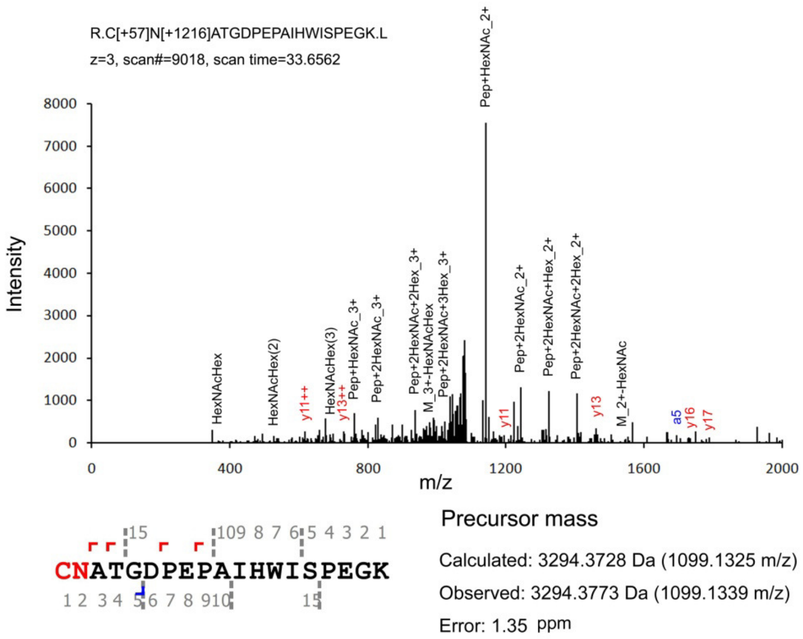
R.C[+57]N[+1216]ATGDPEPAIHWISPEGK.L
z=3, scan#=9018, scan time=33.6562
Intensity
m/z
HexNAcHex
HexNAcHex(2)
HexNAcHex(3)
y11++
y13++
Pep+HexNAc_3+
Pep+2HexNAc_3+
Pep+2HexNAc+2Hex_3+
M_3+-HexNAcHex
Pep+2HexNAc+3Hex_3+
Pep+HexNAc_2+
y11
Pep+2HexNAc_2+
Pep+2HexNAc+Hex_2+
Pep+2HexNAc+2Hex_2+
y13
M_2+-HexNAc
a5
y16
y17
CNATGDPEPAIHWISPEGK
Precursor mass
Calculated: 3294.3728 Da (1099.1325 m/z)
Observed: 3294.3773 Da (1099.1339 m/z)
Error: 1.35 ppm

(c)

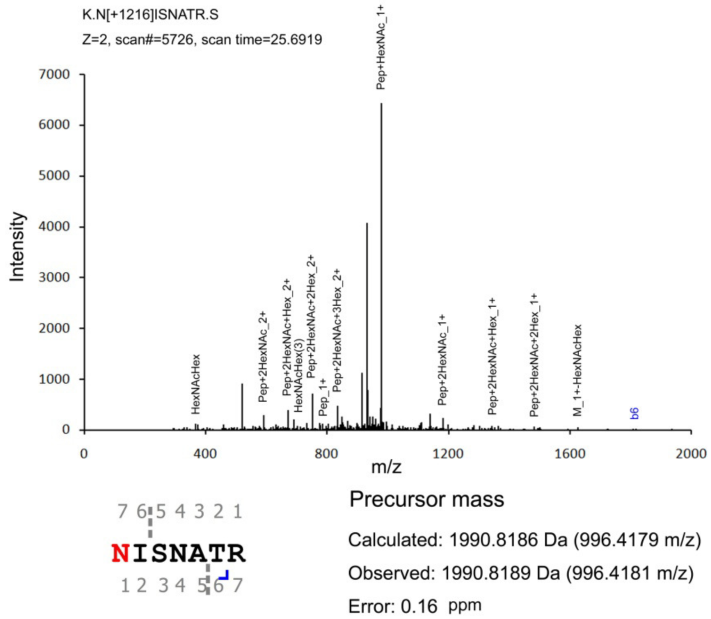

(d)

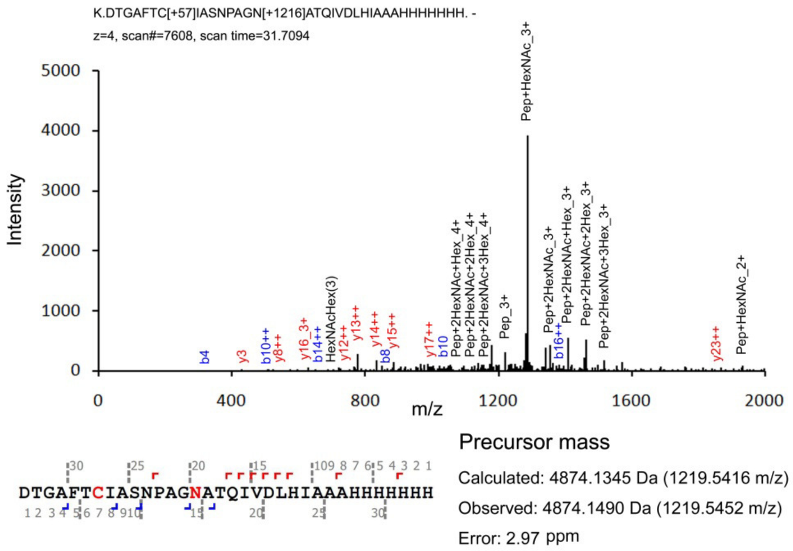

Supplementary Figure 3: Mass spectrometry analysis of "glycan wedge" introduced in SALM5. (a-d) are MS/MS spectra of human SALM5 LRR-Ig mutants: R110N/E160S (a); K309N/R311T (b); L327N (c) and E365N (d). Below each spectrum is the detected peptide sequence with assigned a- (or b-, in blue) and y-ion (in red) fragments. The calculated and observed precursor masses of the peptide are also

shown. For each mutant, a molecular mass change of 1216 Da was observed at residue Asn (in red), indicating the success of introducing an N-linked glycan, GlcNAc2Man5, which is line with the glycosylation mode characterized by HEK293S GnTI^{-} cells. For the mutant R110N/E160S, the predicted glycan at N158 was not detected. In the spectra, Hex represents Glc or Man.

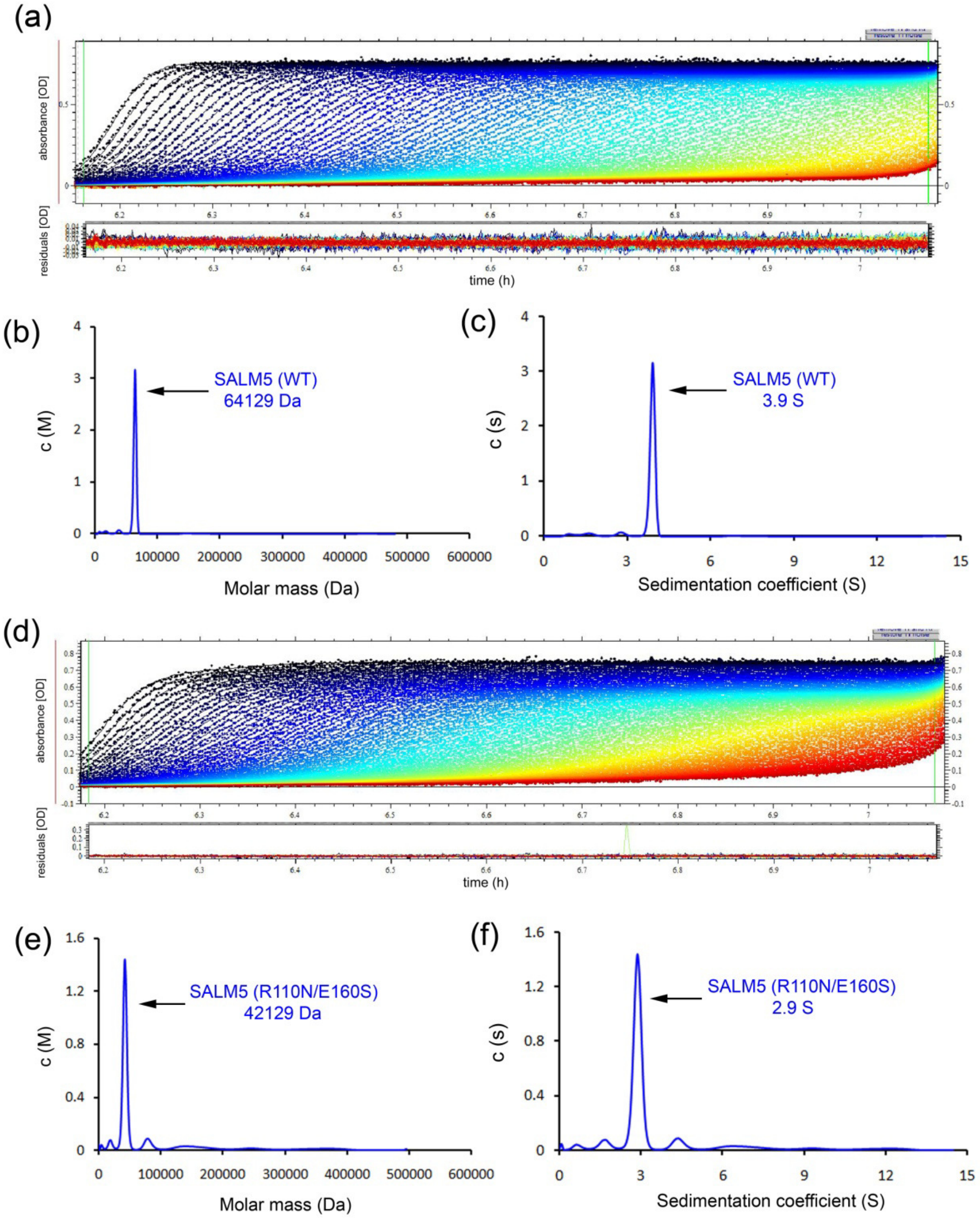

Supplementary Figure 4: Analytical ultracentrifugation studies of human SALM5 LRR-Ig wild type (called SALM5 WT for short) (a-c) and mutant R110N/E160S (d-f). (a) and (d), curves represent absorbance at 280 nm (OD_{280}, along the vertical axis) versus time (in hour, along the horizontal axis) in the upper panels, as well as the residuals of OD_{280} (along the vertical axis) versus time (in hour, along the horizontal axis) in the lower panels; (b) and (e) are curves for molar mass distribution, c(M); (c)

and (f) are curves for sedimentation coefficient distribution, c(s). A caveat should be given here that sedimentation velocity experiment has intrinsic limitations in deducing molecular masses of proteins. However, since there is one dominant species in both SALM5 WT and mutant sample solutions, the c(M) distribution can be derived from the c(s) distribution for molecular mass calculation. The calculated molecular masses corresponding to the main peaks of SALM5 WT and mutant in the curves are about 64.1 kDa and 42.1 kDa, respectively. Likely due to two protomers packing into a side-by-side configuration, the wild-type SALM5 dimer presented an apparent molecular mass less than two times of the theoretical value 43.6 kDa for a wild-type monomer. The theoretical molecular mass of one mutant molecule is 44.8 kDa, since it contains four N-linked glycosylation sites with three (N73, N330 and N339) shown in the crystal structure (Figure 1d) and one (N110) convinced by mass spectrometry (Figure S3a). Therefore, this sedimentation velocity study suggests that the mutant R110N/E160S mainly exists as a monomer in solution.

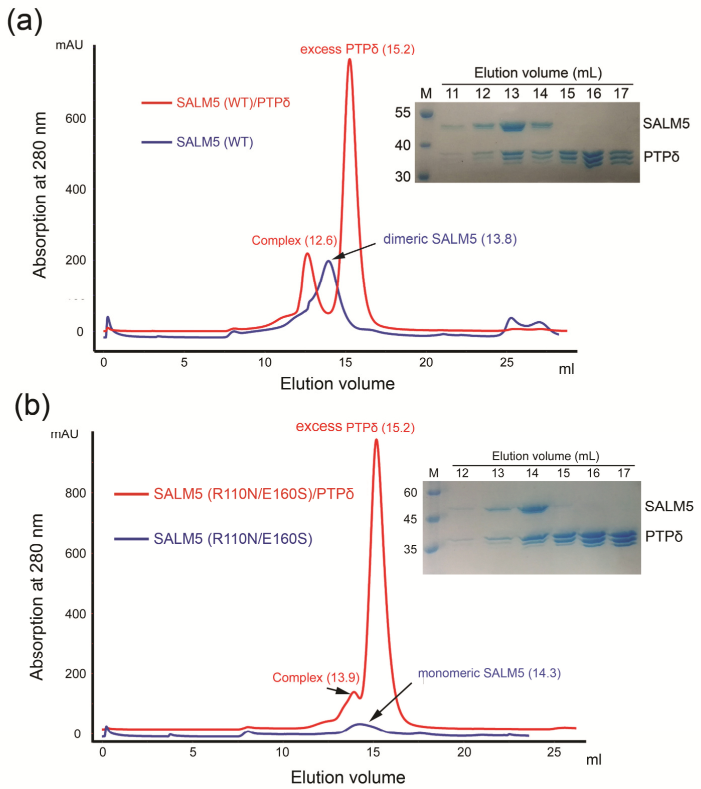

Supplementary Figure 5: Size exclusion chromatography (SEC) analysis of SALM5/PTPδ interaction. (a) SEC profiles of human SALM5 LRR-Ig (labeled as SALM5 for short in this figure) alone (in slate) and in complex with human PTPδ Ig 1-3 (MeA^{-}) (labeled as PTPδ for short in this figure) (in red). (b) SEC profiles of SALM5 R110N/E160S alone (in slate) and in complex with PTPδ (in red). The profiles are accompanied by insets showing SDS-PAGE analysis of peak fractions, and molecular weight markers (kDa) in lane M are labeled. The wild-type SALM5/PTPδ complex eluted at an elution volume of 12.6 ml, while the mutant SALM5/PTPδ complex eluted at 13.9 ml. Under the same purification conditions as used for wild-type SALM5, SALM5 R110N/E160S demonstrated poor stability as

shown by a low and wide peak on the SEC profile. However, the complex between SALM5 R110N/E160S and PTPδ was stable and eluted at fractions 12-14 as resolved by SDS-PAGE analysis.

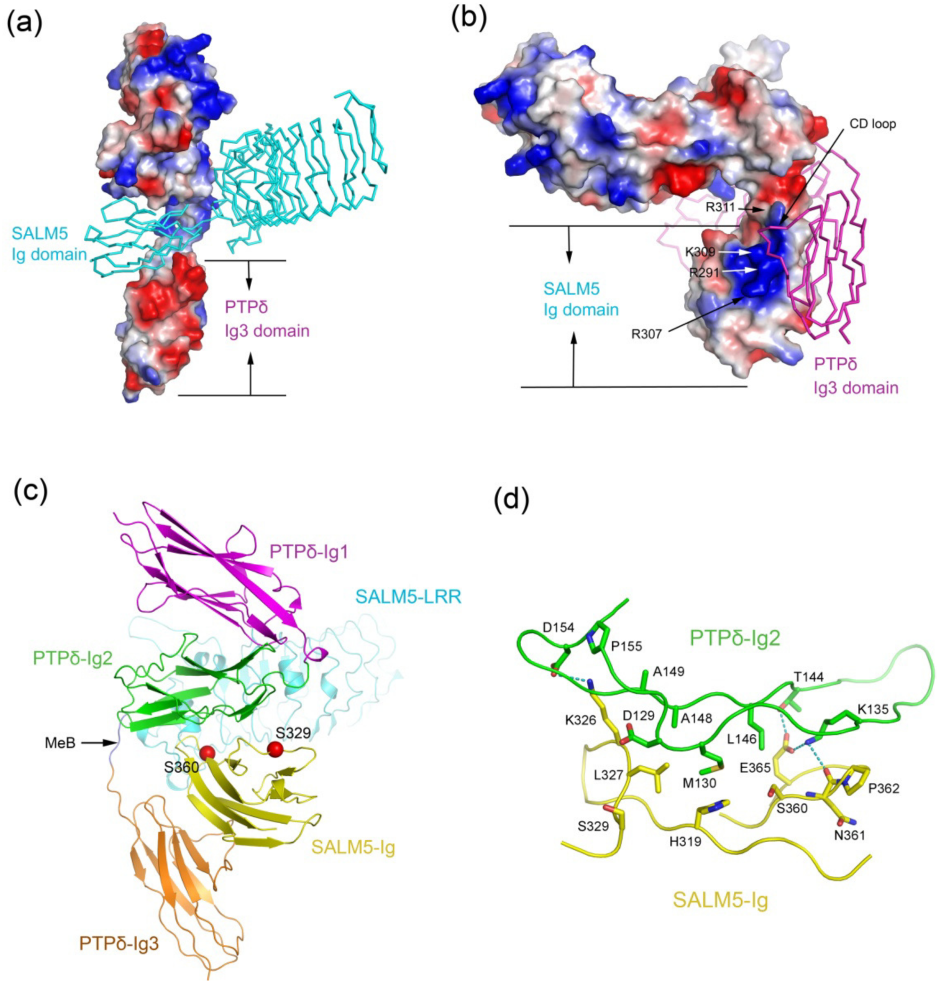

Supplementary Figure 6: Revisting of the SALM5/PTPδ interface. (a-b) Surface electrostatic potential representation of SALM5/PTPδ complex. Both PTPδ (a) and SALM5 (b) are colored by electrostatic potential from red (negatively charged) to blue (positively charged). SALM5 R291, K309 and R311 form a positively charged region on the surface of Ig domain (b). This positive region interacts with the CD loop of PTPδ Ig domain that is negatively charged. (c) Location of S329 and S360 around the SALM5/PTPδ complex interface, with their C_{α} atoms shown in red sphere. (d) Stick representation of residues in the SALM5/PTPδ complex interface, including

SALM5 S329 and S360. Color code for each domain in (c) and (d) is the same as Figure 3a, while (d) has a 180° rotation relative to Figure 3d. SALM5 S329 and S360 are hardly interacting with PTPδ, except that the C_β atom of S360 has a weak van de Waals interaction with PTPδ as shown in Supplementary Table 3.

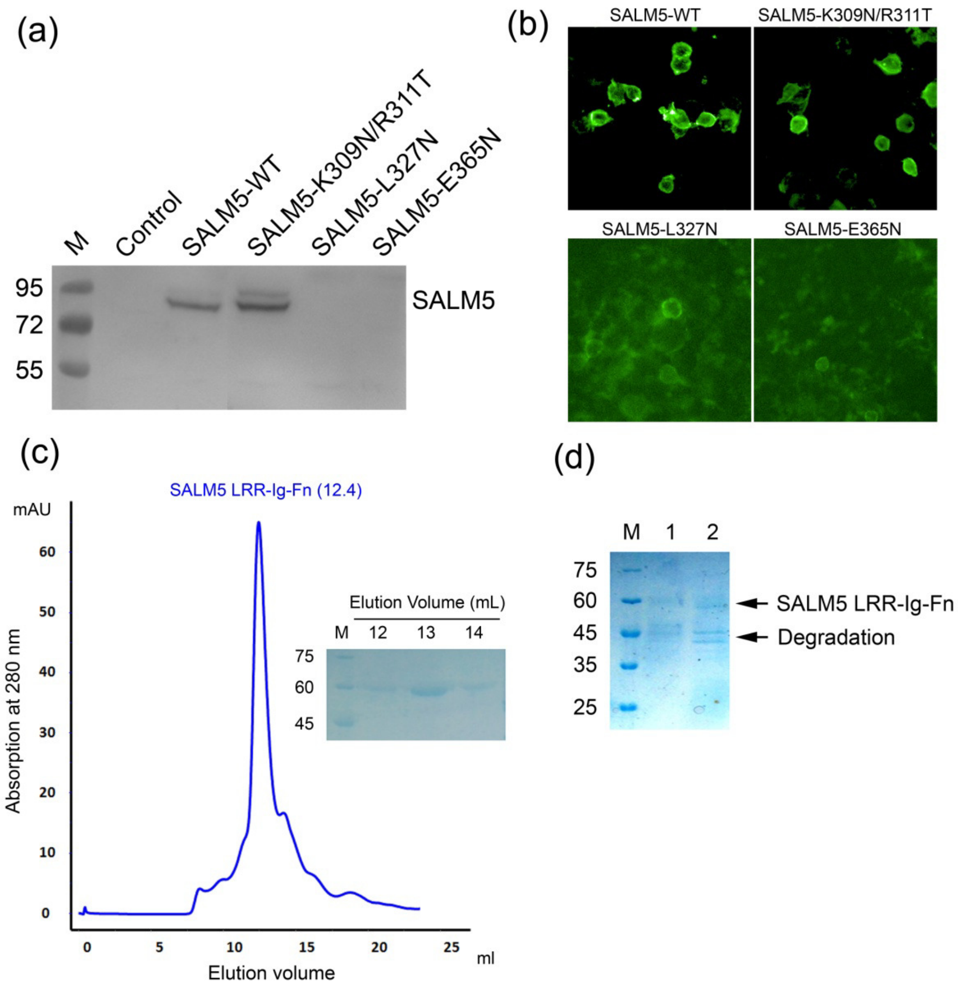

Supplementary Figure 7: Biochemical and cellular characterizations of human SALM5 ectodomain and its mutants. (a) Western-blotting analysis. Lysates of HEK293T cells transfected with plasmids of empty pDisplay vector (control), SALM5 ectodomain (WT) or mutants K309N/R311T, L327N and E365N, were immuno-blotted with mouse serum containing anti-SALM5 polyclonal antibodies. Only WT and K309N/R311T mutant SALM5 were expressed. (b) Representative images of HEK293T cells displaying SALM5 ectodomain (WT) or its mutants K309N/R311T, L372N and E365N that were immuno-stained using anti-HA

antibodies (green). Scale bar, 25 μm. (c) Gel filtration chromatogram and SDS-PAGE analysis of human SALM5 ectodomain (LRR-Ig-Fn). The elution volume was 12.4 ml. (d) Degradation of SALM5. In comparison with the fresh sample in (c), the purified SALM5 ectodomain underwent degradation after 2-day storage at 4 °C in the absence (in lane 1) and presence (in lane 2) of Endo H. Due to degradation that occurred to the recombinant full-length SALM5 ectodomain, we propose that L327N and E365N mutations would likely accelerate such a degradation so that the mutants couldn't be tested out on the transfected HEK293T cells. For (c-d), molecular weight markers (kDa) in lane M are labeled.

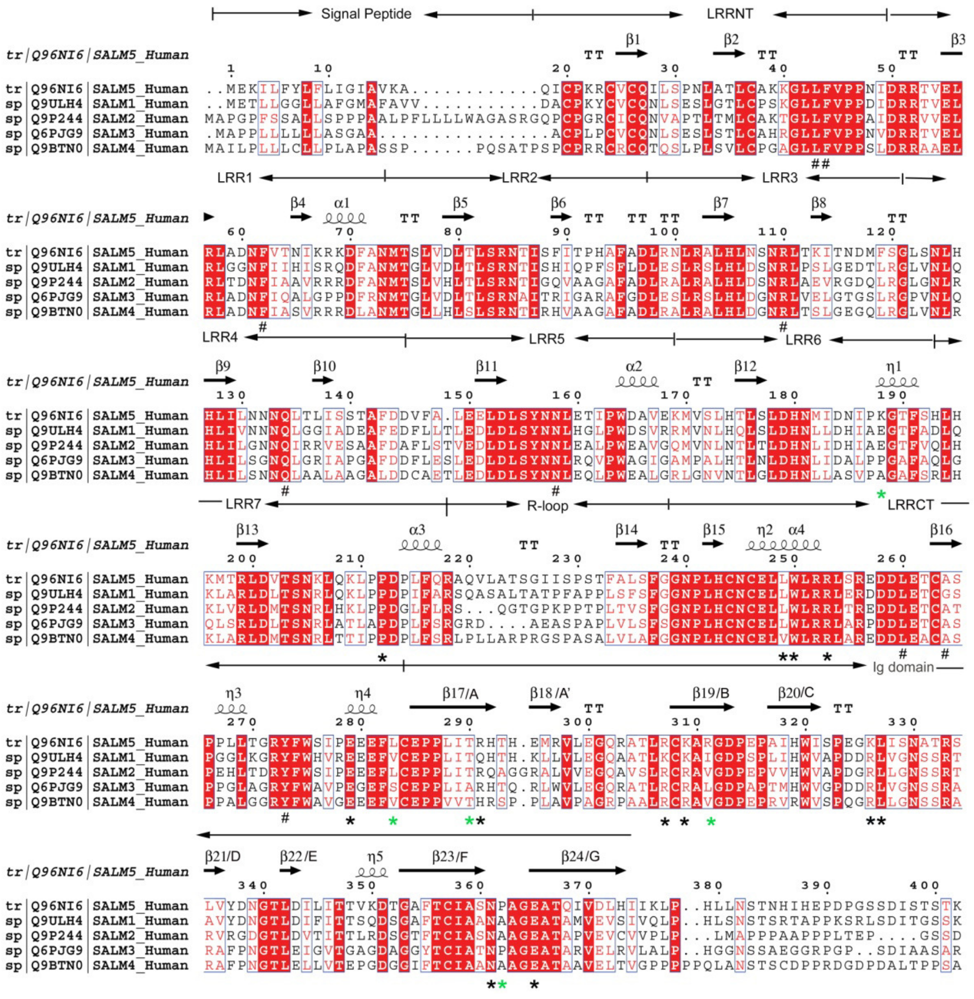

Supplementary Figure 8: Structure-based sequence alignment of human SALMs 1-5. The residues located on the SALM5 dimer interface are labeled with number signs (#). The SALM5 residues involved in PTPδ interaction and conserved in SALM3 are labeled with black asterisks (*). The residues not conserved in SALM3, but involved in PTPδ interaction, are labeled with green asterisks.

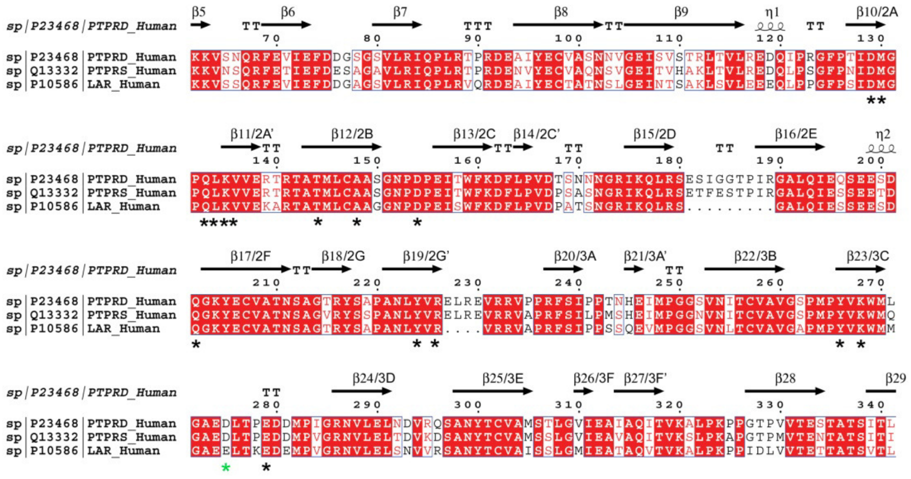

Supplementary Figure 9: Structure-based sequence alignment of human PTPδ (labeled as PTPRD) Ig2-3 and its homologous counterparts in LAR and PTPσ (labeled as PTPRS). The PTPδ residues involved in SALM5 interaction are labeled with black (for absolutely conserved residues) or green (for highly conserved one) asterisks. Numbering for PTPδ residues after S180 herein is larger than that described in the main text by 9. For instance, PTPδ Y224 in this figure is equivalent to Y215 in Figure 3.

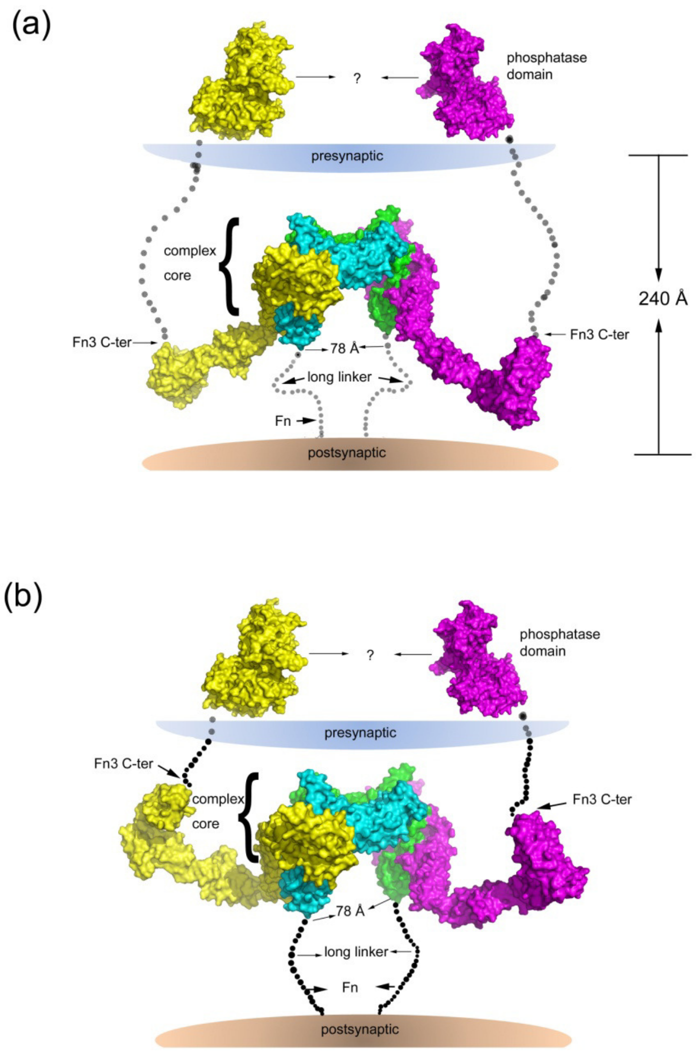

Supplementary Figure 10: A model proposed for SALM5-induced dimerization and activation of PTPδ. The postsynaptic *cis*-SALM5 dimer is indicated in surface representation with its two protomers colored in cyan and green. The C-termini of two SALM5 Ig domains are 78 Å apart. (a) Model for the native PTPδ with eleven domains (including eight Fn domains) in its extracellular region. The estimated distance between the C-termini of two PTPδ Fn domains (modeled following the structure of PTPσ with PDB code 4PBX) is about 250 Å. The remaining five Fn domains of PTPδ and the Fn domain of SALM5 are showed in black dotted lines. (b)

Model for the PTPδ isoform with seven domains (four Fn domains) in its extracellular region. In comparison with the counterpart in the model (a), the PTPδ isoform would adopt a more bent configuration, while the complex core (SALM5 LRR-Ig/PTPδ Ig1-3) would be closer to the presynaptic membrane since SALM5 has an unusual, long flexible linker (~ 50 AA long and rich of serine) connecting its Ig and Fn domains.

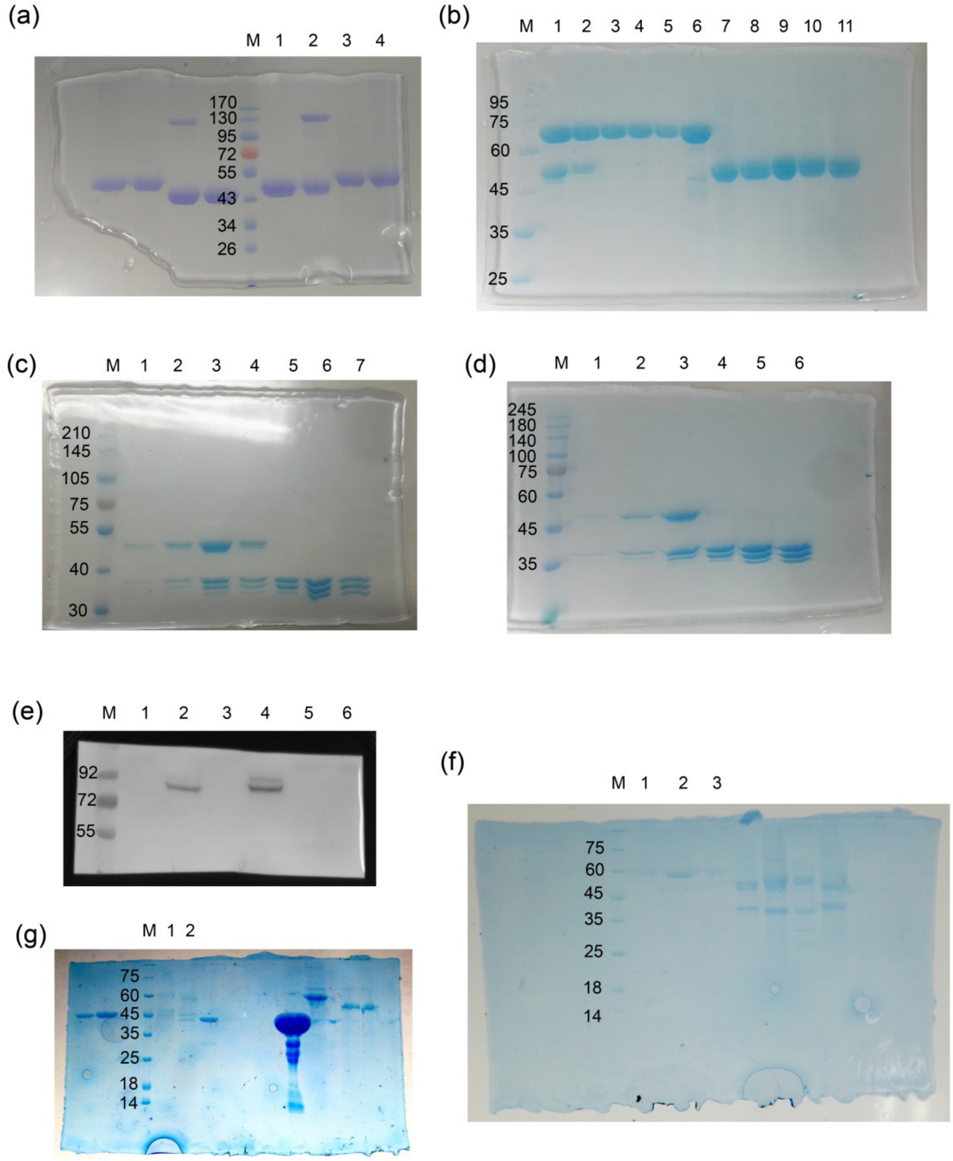

Supplementary Figure 11: Uncropped images of gels and blots with molecular weight markers (kDa) in lane M labeled. (a) SDS-PAGE analysis of the wild-type SALM5 LRR-Ig (lanes 1 and 2) and its R110N/E160S mutant (lanes 3 and 4) in the presence (lanes 1 and 3) or absence (lanes 2 and 4) of BS^3, corresponding to Figure 2d. (b) SDS-PAGE analysis of SALM5 LRR-Ig wild-type and its variants L327N, E365N and K309N/R311T (lanes 1-4) pulled down by Fc-tagged PTPδ Ig1-3 (MeA^-), as well

as proteins (lanes 6-10) used in the pull-down assay, respectively corresponding to the upper and lower panels of Figure 4a. (c) SDS-PAGE analysis of SEC-purified SALM5 LRR-Ig wild-type in complex with PTPδ Ig1-3 (MeA^-), corresponding to the inset of Supplementary Figure 5a. (d) SDS-PAGE analysis of SEC-purified of SALM5 LRR-Ig R110N/E160S mutant in complex with PTPδ Ig1-3 (MeA^-), corresponding to the inset of Supplementary Figure 5b. (e) Western-blotting analysis of HEK293T cells harboring or expressing empty pDisplay vector (control, lane 1), human SALM5 ectodomain wild-type (lane 2) and its variants K309N/R311T (lane 4), L327N (lane 5), and E365N (lane 6), corresponding to Supplementary Figure 7a. (f) SDS-PAGE analysis of SEC-purified human SALM5 ectodomain and other proteins, with lanes 1-3 corresponding to Supplementary Figure 7c. (g) SDS-PAGE analysis of human SALM5 ectodomain in degradation and other proteins, with lanes 1 and 2 corresponding to Supplementary Figure 7d.

Supplementary Tables

Supplementary Table 1 Sequences of primer pairs used in this study

**Construct (residues)**	**Vector**	**Primer sequences**	**Description**
Human SALM5 LRR-Ig (18-374)	BacMam	Forward: 5’-ACGTggatccTCAGATCTGTCCAAAGCGTTGTG-3’<br>Reverse: 5’-CGATgcggccgcTATATGAAGATCCACTATTTGTGTTGCTTCC-3’	Single-step PCR
Human SALM5 ectodomain (18-526)	BacMam	Forward: 5’-ACGTggatccTCAGATCTGTCCAAAGCGTTGTG-3’<br>Reverse: 5’-CGATgcggccgcGGTGCCTCCCAAAAACTGG-3’	Single-step PCR
Human PTPδ Ig1-3 (21-320)	BacMam	Forward: 5’-ACGTctcgagAGGAGACACCTCCAAGGTTTACAC-3’<br>Reverse: 5’-CGATgcggccgcTTTGACAGTGATCTGTGCTATTGC-3’	Single-step PCR
Human PTPδ Ig1-3 (MeA^{-})	BacMam	Forward-1: 5’-ACGTctcgagAGGAGACACCTCCAAGGTTTACAC-3’<br>Reverse-1: 5’-CGATgcggccgcTTTGACAGTGATCTGTGCTATTGC-3’<br>Forward-2: 5’-CAGTTACGATCAGGAGCCCTTCAGATTGAGCAG-3’<br>Reverse-2: 5’-CTGAAGGGCTCCTGATCGTAACTGCTTAATACGACCATTG-3’	Two-step PCR [a]
Human PTPδ Ig1-3 (MeB^{-})	BacMam	Forward-1: 5’-ACGTctcgagAGGAGACACCTCCAAGGTTTACAC-3’<br>Reverse-1: 5’-CGATgcggccgcTTTGACAGTGATCTGTGCTATTGC-3’<br>Forward-2: 5’-CAATTTATATGTCAGAGTTCGCCGTGTCCCAC-3’<br>Reverse-2: 5’-GGACACGGCGAACTCTGACATATAAATTGGCAGGAGCG-3’	Two-step PCR [a]
Human PTPδ Ig1-3 (MeA^{-}/MeB^{-})	BacMam	Forward-1: 5’-ACGTctcgagAGGAGACACCTCCAAGGTTTACAC-3’<br>Reverse-1: 5’-CGATgcggccgcTTTGACAGTGATCTGTGCTATTGC-3’<br>Forward-2: 5’-CAGTTACGATCAGGAGCCCTTCAGATTGAGCAG-3’<br>Reverse-2: 5’-CTGAAGGGCTCCTGATCGTAACTGCTTAATACGACCATTG-3’<br>Forward-3: 5’-CAATTTATATGTCAGAGTTCGCCGTGTCCCAC-3’<br>Reverse-3: 5’-GGACACGGCGAACTCTGACATATAAATTGGCAGGAGCG-3’	Two-step PCR [b]
Human SALM5 LRR-Ig (R110N/E160S)	BacMam	Forward-1: 5’-ACGTggatccTCAGATCTGTCCAAAGCGTTGTG-3’<br>Reverse-1: 5’-CGATgcggccgcTATATGAAGATCCACTATTTGTGTTGCTTCC-3’<br>Forward-2:<br>5’-GCATTTGAATAGCAACAACTTGACTAAAATTACAAATGATATGTTCAGTGGTC-3’<br>Reverse-2: 5’-CATTTGTAATTTTAGTCAAGTTGTTGCTATTCAAATGCAAAGCCCTC-3’<br>Forward-3: 5’-CTGTCCTATAATAATCTAAGCACCATTCCTTGGGATGCTGTTG-3’<br>Reverse-3: 5’-CCAAGGAATGGTGCTTAGATTATTATAGGACAGATCCAGCTCCTC-3’	Two-step PCR [b]
Human SALM5 LRR-Ig (L327N)	BacMam	Forward-1: 5’-ACGTggatccTCAGATCTGTCCAAAGCGTTGTG-3’<br>Reverse-1: 5’-CGATgcggccgcTATATGAAGATCCACTATTTGTGTTGCTTCC-3’<br>Forward-2: 5’-CCTGAAGGGAAGAACATTTCAAATGCAACAAGATCTCTGGTG-3’<br>Reverse-2: 5’-GTTGCATTTGAAATGTTCTTCCCTTCAGGAGAAATCCAGTG-3’	Two-step PCR [a]

(*to be continued*)

Human SALM5 LRR-Ig (K309N/R311T)	BacMam	Forward-1: 5’-ACGTggatccTCAGATCTGTCCAAAGCGTTGTG-3’<br>Reverse-1: 5’-CGATgcggccgcTATATGAAGATCCACTATTTGTGTTGCTTCC-3’<br>Forward-2: 5’-GCAACGCCACCGGAGACCCTGAGCCTGC-3’<br>Reverse-2: 5’-GTCTCCGGTGGCGTTGCACCTCAGTGTTGCCC-3’	Two-step PCR [a]
Human SALM5 LRR-Ig (E365N)	BacMam	Forward-1: 5’-ACGTggatccTCAGATCTGTCCAAAGCGTTGTG-3’<br>Reverse-1: 5’-CGATgcggccgcTATATGAAGATCCACTATTTGTGTTGCTTCC-3’<br>Forward-2: 5’-CCTGAAGGGAAGAACATTTCAAATGCAACAAGATCTCTGGTG-3’<br>Reverse-2: 5’-GTTGCATTTGAAATGTTCTTCCCTTCAGGAGAAATCCAGTG-3’	Two-step PCR [a]
Fc-tagged Human PTPδ Ig1-3 (MeA$^-$)	BacMam (with Fc)	Forward: 5’-ACGTctcgagAGGAGACACCTCCAAGGTTTACAC-3’<br>Reverse: 5’-CGATgaattcTCTTTGACAGTGATCTGTGCTATTGC-3’	Single-step PCR
Human SALM5 ectodomain (18-526)	pDisplay	Forward: 5’-ATCGggcccagccggccCAGATCTGTCCAAAGCGTTGTG-3’<br>Reverse: 5’-CGATccgcggGGTGCCTCCCAAAAACTGG-3’	Single-step PCR
Human SALM5 ectodomain (R110N/E160S)	pDisplay	Forward-1: 5’-ATCGggcccagccggccCAGATCTGTCCAAAGCGTTGTG-3’<br>Reverse-1: 5’-CGATccgcggGGTGCCTCCCAAAAACTGG-3’<br>Forward-2:<br>5’-GCATTTGAATAGCAACAACTTGACTAAAATTACAAATGATATGTTCAGTGGTC-3’<br>Reverse-2: 5’-CATTTGTAATTTTAGTCAAGTTGTTGCTATTCAAATGCAAAGCCCTC-3’<br>Forward-3: 5’-CTGTCCTATAATAATCTAAGCACCATTCCTTGGGATGCTGTTG-3’<br>Reverse-3: 5’-CCAAGGAATGGTGCTTAGATTATTATAGGACAGATCCAGCTCCTC-3’	Two-step PCR [b]
Human SALM5 ectodomain (K309N/R311T)	pDisplay	Forward-1: 5’-ATCGggcccagccggccCAGATCTGTCCAAAGCGTTGTG-3’<br>Reverse-1: 5’-CGATccgcggGGTGCCTCCCAAAAACTGG-3’<br>Forward-2: 5’-GCAACGCCACCGGAGACCCTGAGCCTGC-3’<br>Reverse-2: 5’-GTCTCCGGTGGCGTTGCACCTCAGTGTTGCCC-3’	Two-step PCR [a]
Human SALM5 ectodomain (L327N)	pDisplay	Forward-1: 5’-ATCGggcccagccggccCAGATCTGTCCAAAGCGTTGTG-3’<br>Reverse-1: 5’-CGATccgcggGGTGCCTCCCAAAAACTGG-3’<br>Forward-2: 5’-CCTGAAGGGAAGAACATTTCAAATGCAACAAGATCTCTGGTG-3’<br>Reverse-2: 5’-GTTGCATTTGAAATGTTCTTCCCTTCAGGAGAAATCCAGTG-3’	Two-step PCR [a]
Human SALM5 ectodomain (E365N)	pDisplay	Forward-1: 5’-ATCGggcccagccggccCAGATCTGTCCAAAGCGTTGTG-3’<br>Reverse-1: 5’-CGATccgcggGGTGCCTCCCAAAAACTGG-3’<br>Forward-2: 5’-CCTGAAGGGAAGAACATTTCAAATGCAACAAGATCTCTGGTG-3’<br>Reverse-2: 5’-GTTGCATTTGAAATGTTCTTCCCTTCAGGAGAAATCCAGTG-3’	Two-step PCR [a]
Human PTPδ Ig1-3 (MeA$^-$)	pDisplay	Forward-1: 5’-ATCGggcccagccggccGAGACACCTCCAAGGTTTACAC-3’<br>Reverse-1: 5’-CGATccgcggTTTGACAGTGATCTGTGCTATTGC-3’<br>Forward-2: 5’-CAGTTACGATCAGGAGCCCTTCAGATTGAGCAG-3’<br>Reverse-2: 5’-CTGAAGGGCTCCTGATCGTAACTGCTTAATACGACCATTG-3’	Two-step PCR [a]

(a) For the first round of PCR, primer sets Forward-1/Reverse-2 and Forward-2/Reverse-1 were used for amplifying cDNA. For the second round of PCR, the primer set Forward-1/Reverse-1 was used with gel-purified PCR products from the first round of PCR as a template.

(b) For the first round of PCR, primer sets Forward-1/Reverse-2, Forward-2/Reverse-3 and Forward-3/Reverse-1

were used for amplifying cDNA. For the second round of PCR, the primer set Forward-1/Reverse-1 was used with gel-purified PCR products from the first round of PCR as a template.

Supplementary Table 2 Contacting residue pairs between two monomers of a SALM5 dimer[#]

atom in monomer 1	atom in monomer 2	distance (Å)
[ARG 23 NH2]	[THR 262 OG1]	3.0
[ARG 23 NH1]	[GLY 271 O]	2.6
[ARG 23 CD]	[TYR 273 OH]	3.1
[LYS 39 O]	[LEU 260 CD1]	3.2
[LYS 40 C]	[LEU 260 CD1]	3.7
[LYS 40 O]	[TYR 273 OH]	2.7
[GLY 41 CA]	[LEU 260 O]	4.0
[GLY 41 O]	[THR 262 OG1]	3.3
[LEU 43 CD1]	[ASN 240 O]	3.8
[LEU 43 CD1]	[THR 262 O]	3.6
[LEU 43 CD1]	[ALA 264 N]	4.0
[PHE 44 CE2]	[THR 270 CG2]	3.8
[PHE 62 CE1]	[SER 204 CB]	3.5
[PHE 62 CE1]	[GLY 239 CA]	3.5
[THR 64 OG1]	[LYS 206 NZ]	3.2
[THR 86 CG2]	[SER 204 OG]	3.2
[ARG 110 NH2]	[ASN 157 O]	2.5
[ARG 110 NH1]	[ASN 158 OD1]	2.9
[ARG 110 NH2]	[HIS 180 O]	3.1
[ARG 110 CD]	[MET 182 CE]	3.6
[GLN 134 CG]	[GLN 134 OE1]	3.6
[GLN 134 OE1]	[ASN 158 ND2]	2.5
[ASN 157 O]	[ARG 110 NH2]	2.6
[ASN 158 OD1]	[ARG 110 NH1]	2.9
[ASN 158 ND2]	[GLN 134 OE1]	2.5
[HIS 180 O]	[ARG 110 NH2]	3.1
[MET 182 CE]	[ARG 110 CD]	3.6
[SER 204 CB]	[PHE 62 CE1]	3.5
[SER 204 OG]	[THR 86 CG2]	3.2
[LYS 206 NZ]	[THR 64 OG1]	3.2
[GLY 239 CA]	[PHE 62 CE1]	3.5
[ASN 240 O]	[LEU 43 CD1]	3.8
[LEU 260 CD1]	[LYS 39 O]	3.2
[LEU 260 CD1]	[LYS 40 C]	3.7
[LEU 260 O]	[GLY 41 CA]	4.0
[THR 262 OG1]	[ARG 23 NH2]	3.0
[THR 262 OG1]	[GLY 41 O]	3.3
[THR 262 O]	[LEU 43 CD1]	3.6
[ALA 264 N]	[LEU 43 CD1]	4.0
[THR 270 CG2]	[PHE 44 CE2]	3.8
[GLY 271 O]	[ARG 23 NH1]	2.6
[TYR 273 OH]	[ARG 23 CD]	3.1
[TYR 273 OH]	[LYS 40 O]	2.7

[#]The atoms that form the closest contact between the particular pair of residues and the corresponding distance are listed. The distance values are calculated using CNS.

Supplementary Table 3 Contacting residue pairs between SALM5 and PTPδ[#]

	atom in SALM5	atom in PTPδ	distance (Å)
**Site-I**			
	[LYS 188 NZ]	[GLN 133 OE1]	3.1
	[PRO 212 CG]	[TYR 215 OH]	3.7
	[CYS 246 CB]	[VAL 136 CG2]	3.8
	[LEU 249 CD2]	[ARG 217 CZ]	3.9
	[TRP 250 CB]	[TYR 215 OH]	3.3
	[ARG 253 NH1]	[GLN 193 OE1]	3.4
	[ARG 253 NH2]	[VAL 216 O]	3.1
	[ARG 253 NH2]	[ARG 217 CB]	3.5
	[GLU 279 O]	[ARG 217 NH2]	2.4
	[GLU 280 CA]	[ARG 217 NH2]	3.5
	[GLU 280 O]	[ARG 220 CD]	3.6
	[PHE 282 O]	[ARG 217 NH1]	2.8
	[LEU 283 CD2]	[ARG 220 NH1]	3.4
**Site-II**			
	[LEU 288 CD1]	[MET 296 CB]	3.7
	[LEU 288 CD2]	[SER 297 O]	3.6
	[THR 290 O]	[LYS 259 NZ]	3.1
	[THR 290 CB]	[GLU 270 CG]	3.7
	[THR 290 CG2]	[MET 273 SD]	3.5
	[THR 290 O]	[MET 296 SD]	3.5
	[ARG 291 NE]	[ASP 266 OD2]	3.5
	[ARG 291 NH1]	[PRO 269 CA]	3.8
	[LYS 309 C]	[GLU 270 OE2]	3.1
	[ALA 310 N]	[GLU 270 OE2]	3.0
	[ARG 311 NH2]	[TYR 257 CE2]	3.3
	[ARG 311 NH1]	[GLU 270 O]	3.3
	[ARG 311 NH1]	[MET 273 CB]	3.8
**Site-III**			
	[HIS 319 ND1]	[MET 130 SD]	3.1
	[LYS 326 CD]	[ALA 149 O]	3.4
	[LYS 326 NZ]	[ASP 154 OD1]	3.2
	[LYS 326 NZ]	[PRO 155 CD]	3.8
	[LEU 327 CD1]	[ASP 129 CB]	3.5
	[LEU 327 CD1]	[MET 130 CE]	3.3
	[LEU 327 CD1]	[ALA 148 CB]	3.6
	[SER 360 CB]	[LEU 146 CD1]	3.7
	[ASN 361 O]	[LYS 135 NZ]	3.0
	[PRO 362 CA]	[LEU 134 O]	3.1
	[PRO 362 O]	[LYS 135 CA]	3.3
	[PRO 362 O]	[VAL 136 N]	3.1
	[GLU 365 OE1]	[LYS 135 NZ]	3.0
	[GLU 365 OE2]	[THR 144 OG1]	2.4
	[GLU 365 CD]	[LEU 146 CD2]	3.7

[#]The atoms that form the closest contact between the particular pair of residues and the corresponding distance are listed. The distance values are calculated using CNS.
